# Influence of Adaptive Gap Control Mechanism and Tool Electrodes on Machining Titanium (Ti-6Al-4V) Alloy in EDM Process

**DOI:** 10.3390/ma15020513

**Published:** 2022-01-10

**Authors:** Shoufa Liu, Muthuramalingam Thangaraj, Khaja Moiduddin, Abdulrahman M. Al-Ahmari

**Affiliations:** 1 School of Mechanical Engineering, Xijing University, Xi’an 710123, China; shoufaliu2000@sina.com; 2 Department of Mechatronics Engineering, SRM Institute of Science and Technology, Kattankulathur 603203, India; 3 Advanced Manufacturing Institute, King Saud University, Riyadh 11421, Saudi Arabia; 4 Raytheon Chair for Systems Engineering (RCSE Chair), Advanced Manufacturing Institute, King Saud University, Riyadh 11421, Saudi Arabia; alahmari@ksu.edu.sa

**Keywords:** discharge, energy, machining, gap control, tool electrodes

## Abstract

Titanium alloy is widely used for orthodontic technology and easily machined using the EDM process. In the EDM process, the workpiece and tool electrode must be separated by a continuous air gap during the machining operation to generate discharge energy in this method. In the present study, an endeavor was made to analyze the effects of a servo feed air gap control and tool electrode in the EDM process. The developed mechanical setup consists of a linear action movement with zero backlash along the X-axis, which can be controlled up to 0.03 mm. It was observed that the suggested air gap control scheme can enhance the servo feed mechanism on a machining titanium alloy. A tungsten carbide electrode can enhance the surface measures owing to its ability to produce tiny craters with uniform distribution. Since it produces a little crater and has a higher melting point, a tungsten carbide electrode can create lesser surface roughness than a copper tool and brass tool electrode.

## 1. Introduction

**Figure 1:**
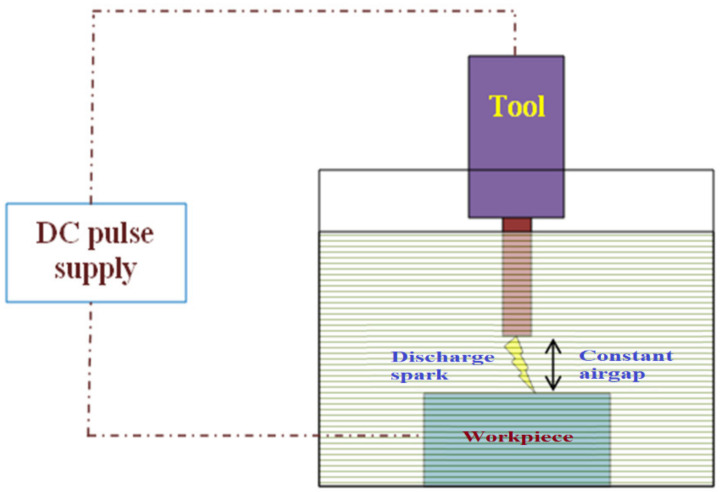
Schematic representation of the EDM process.

Titanium alloy (Ti-6Al-4V) is mostly used in the orthodontic field due to its lower weight and higher corrosion resistance [1]. It can be easily machined by the electrical discharge machining (EDM) and electrochemical machining (ECM) processes [2]. Since the ECM process may affect the top layer of the machined surface, EDM is normally utilized for machining such alloy [3]. It is a novel type of machining in which material is removed by applying regulated electrical pulses between the tool electrode and the workpiece specimen, as illustrated in Figure 1 [4,5]. Since the electrical discharge energy can be controlled by an air gap or stand-off distance (SOD) across the machining zone, the distance between the tool and the electrode should remain constant by implementing a servo tool feed mechanism in the EDM process [6]. As the base metal erodes and the spark gap increases, the electrode should automatically be decreased [7,8]. Various approaches are being employed to implement the servo feed mechanism. However, the most utilized servo feed mechanisms are based on a voltage-sensing mechanism in the EDM process [9]. The variation in the air gap mechanism at the machining zone during the machining process can affect the machinability in the EDM process. The present approaches have mostly dealt with either the signals from the voltage signals or the current signals. If both the signals would be considered for the servo mechanism, the efficacy of the servo mechanism could be improved under less signal disturbance. It has also been observed that enhancement is still needed to obtain a constant stand-off distance. Hence, the SOD needs to be monitored throughout the process to enhance the performance measures. A servo mechanism was designed to obtain a constant SOD with the potential difference and current flow as controlling parameters [10,11]. Since the electrical conductive electrode and specimen were separated by a dielectric medium, electrical discharge happened across the machining zone [12]. The coated tool affects the performance measures in EDM [13]. In the EDM process, the conductivity of the tool electrode can alter the surface morphology of the machined surface. The diffused electrode can significantly minimize the specimen’s surface roughness [14]. The optimal selection of the electrode may reduce tool wear with better surface quality [15]. The cryogenically treated tool electrode can improve the tool electrode’s surface hardness. This increases the rate of material removal while maintaining a high level of surface quality [16]. The melting point of the tool electrode has an effect on its resolidification. It is capable of adjusting the thickness of the white layer applied to the machined surface [17]. The composite electrode can alter the surface measures in the EDM process [18]. The size and shape of the tool electrode can change the material removal mechanism in the process [19]. The heat-treated tool electrode can change the surface topography of the machined specimen [20]. The electrical conductivity of the tool electrode can modify the energy developed during the machining process [21]. The thermal characteristics of the electrodes influence the surface morphology in the EDM process [22]. The tool wear of the electrode can also influence the surface morphology of the specimen in the process [23,24]. The physical characteristics of the electrode in the process could alter the quality measures of the machining process in the machining engineering specimen. From the literature survey, it was found that merely a passing glance was provided to investigate the effect of the adaptive gap control mechanism on the enhancement of the surface quality by reducing the arcing effect. Additionally, it was discovered that just a few studies were conducted to examine the effect of the tool electrode on the surface quality during the die sinking EDM process when machining a titanium alloy. As a result, the current investigation was conducted. The purpose of this work is to develop a gap voltage sensing-based servo feed control system for efficient monitoring of the electrical discharge machining process. Based on the research gap identified, the below-mentioned objectives are made.

- To implement an adaptive gap control mechanism to reduce the arcing effect while machining a titanium alloy;
- To investigate the influence of the adaptive gap control mechanism on the enhancement of the surface quality;
- To conduct an analysis of the tool electrode’s impacts on the surface roughness and morphology in the die sinking EDM process.

## 2. Materials and Methods

Titanium alloy (Ti-6Al-4V) is mostly used in manufacturing industries due to its lighter weight and higher corrosion resistance. Therefore, it was used as specimen material in the present study. Rectangular-shaped duplex annealed workpiece specimens (15 mm × 15 mm) with a length of 20 mm were used as workpiece. The specimens were involved with developed EDM drilling to create a 2 mm blind hole. The specimens were machined under the developed algorithms and different tool electrodes.

### 2.1. Design of EDM Process Arrangement

**Figure 2:**
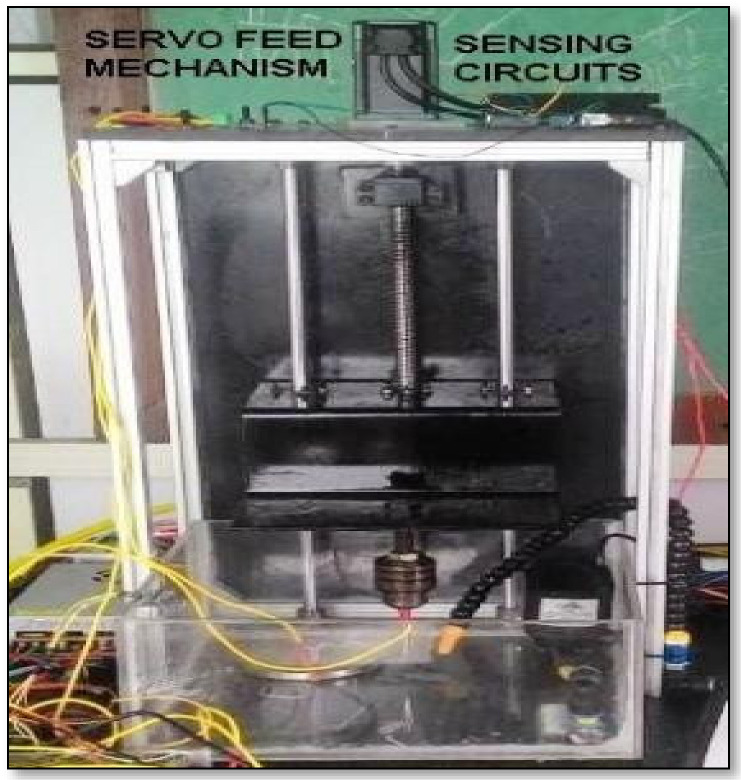
EDM arrangement.

**Figure 3:**
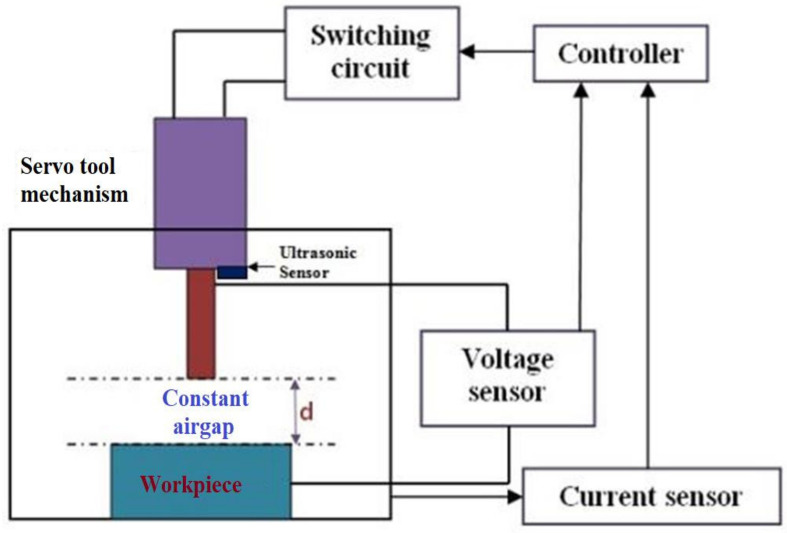
Design of the proposed spark control gap.

The experiments were conducted using an EDM arrangement, as shown in Figure 2. The MAX308 function generator IC package was used to produce a signal with a frequency range of 20 MHz. An IRF540N Power MOSFET (Fairchild Semiconductor, San Jose, CA, USA) was utilized as a gadget for switching in the EDM arrangement with TC2246 as a MOSFET driver circuit. Isolation circuit and short circuit protection was employed to avoid damage to the controller from the high current machining side. An ultrasonic sensor was employed to modify the level of the tool holder. Electrical discharge generated the spark energy in the EDM process. The actuation of the servo mechanism to utilize maximum spark timing used the signals from the current sensor and voltage sensor. However, the signals from the sensors had to be refined for better enhancement of the system, as shown in Figure 3. The voltage sensor was connected across the tool electrode and workpiece. The current sensor was connected in a series with the tool electrode. Due to the capacitive nature of the electrical discharge, the distance between the tool and the workpiece can affect the spark energy per Equation (1):

(1) $$C=\frac{ƐA}{d}$$

where *C* denotes the capacitance, *P* denotes the electrical permittivity, A denotes the tool electrode’s cross-section area, and *d* is the distance between the tool electrode and the workpiece.

### 2.2. Design of the Servo Tool Feed Control

The potential difference between the tool electrode and the workpiece specimen was determined using the potential divider principle and a voltage measuring equipment [25,26]. In this investigation, the gap between the workpiece and the tool electrode was kept between 0.01 mm and 0.1 mm. The response time was based on the PWM signals applied across the machining zone, controllers, and sensors’ compatibility. The response time of the proposed method was found to be 0.1 microsecond. The flowing current was sensed using an ACS712 Hall effect sensor. The voltage and current waveforms were derived from NI-based DSOX3012T (70 MHz, Keysight Technologies, Bangalore, India), a two-channel digital storage oscilloscope with an inbuilt 100 MHz function generator. The Z-axis tool movement was obtained using an RMS-110 hybrid servo stepper motor along a linear ball screw mechanism. The tool holder was positioned using an Ultrasonic HCSR04 sensor. It was placed at the bottom of the tool post, as shown in Figure 3. The ultrasonic sensor can compute the distance travelled between two reference points using the speed of the ultrasonic waves and the time travelled to reach the point. The distance travelled by the tool holder was calibrated with the tool movement time using the servo feed control in the present study.

### 2.3. Design of Experiments

Numerous parameters are involved in the EDM process. Gap voltage (V), discharge current (I), and duty factor (DF) were chosen as the study’s input parameters since they have the greatest influence on the EDM process. Copper (Cu), brass, and tungsten carbide (WC) were used as tool electrode materials in this work to machine titanium alloy specimens with a thickness of 5 mm utilizing the EDM process. Yellow brass (Copper-65% and Zinc-35%) was used in the present study. The physical properties of the tool electrodes are shown in Table 1.

EDM drilling was used to create a 2 mm blind hole. Due to the fact that the machining process comprises three input variables with three interactions (V&I, I&DF, and DF&V), the L_27_ orthogonal table was chosen in accordance with the Taguchi design of experiments, as shown in Table 2. Due to the fact that the experiments must be conducted at lower, medium, and higher levels of electrical energy, open-circuit voltages of 40, 60, and 80 V with duty factors of 0.4, 0.6, and 0.8 were chosen. Amounts of 9, 12, and 15 A were chosen as the highest currents. The average surface roughness (R_a_), which is generally reported in m, is an excellent indicator of the EDM product’s surface quality. The R_a_ was determined in this study utilizing a SE1200 Kosaka lab surfcoder surface roughness tester (Tokyo, Japan). The cutoff length was set at 0.8 mm, and the evaluation length at 2.4 mm. A Keyence VHX-2000 microscope (Chennai, India) was used to acquire a three-dimensional picture of the machined surface produced by the EDM process utilizing conventional and customized pulse generators.

## 3. Results and Discussion

### 3.1. Waveform Analysis

**Figure 4:**
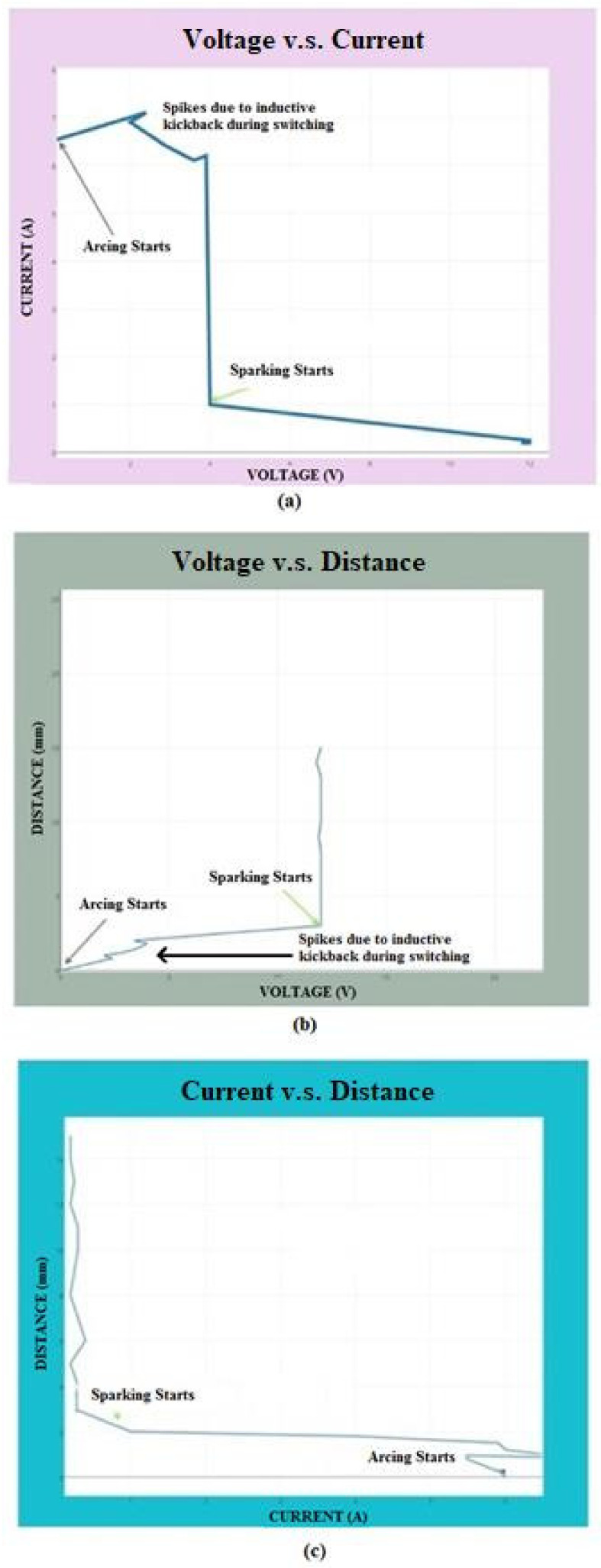
Waveform analysis: (**a**) voltage vs. current, (**b**) voltage vs. distance, (**c**) current vs. distance.

In the EDM process, sparking is a required effect to maintain a better machining mechanism, whereas arcing can produce an undesirable effect. Figure 4 shows the voltage between the tool electrode and the workpiece specimen versus the flowing current. It was observed that arcing happens at a lower voltage, whereas sparking happens at a considerable voltage and current, as shown in Figure 4.

**Figure 5:**
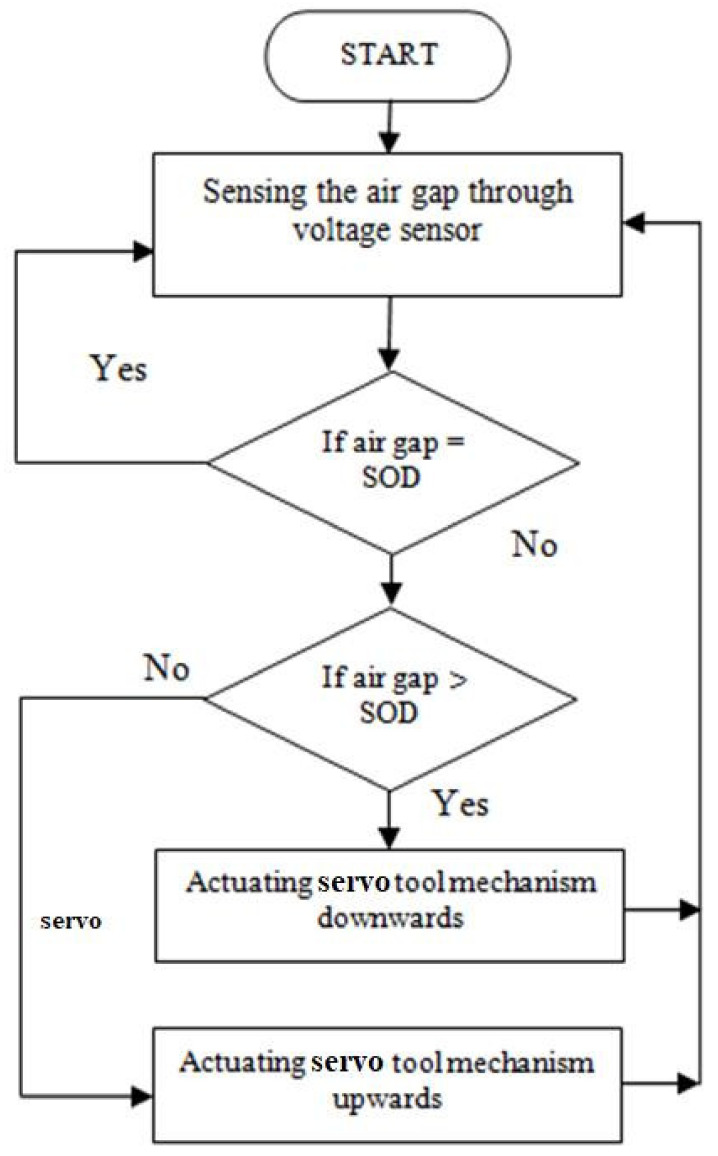
Flow chart for the servo tool feed control algorithm.

In capacitance, the voltage between the electrodes is directly proportional to the distance between them. Hence, the minimum voltage can produce an arcing effect. The distance between the tool electrode and the workpiece can make either sparking or arcing produced [27]. Spikes were observed owing to the inductive kickback occurrence happening during switching from arcing to sparking and vice versa. A distance shorter than the constant SOD may produce an arcing effect. The distance between the specimen and the electrode was measured using signals from the voltage and current sensor. It was compared with the SOD. According to the error values, necessary actions were performed, as mentioned in Figure 5. The efficiency of the proposed gap sensing mechanism was compared with those in previous works [1,4]. It was inferred that the arcing effect was considerably reduced owing to the efficient switching ability.

### 3.2. Pulse Form Analysis

**Figure 6:**
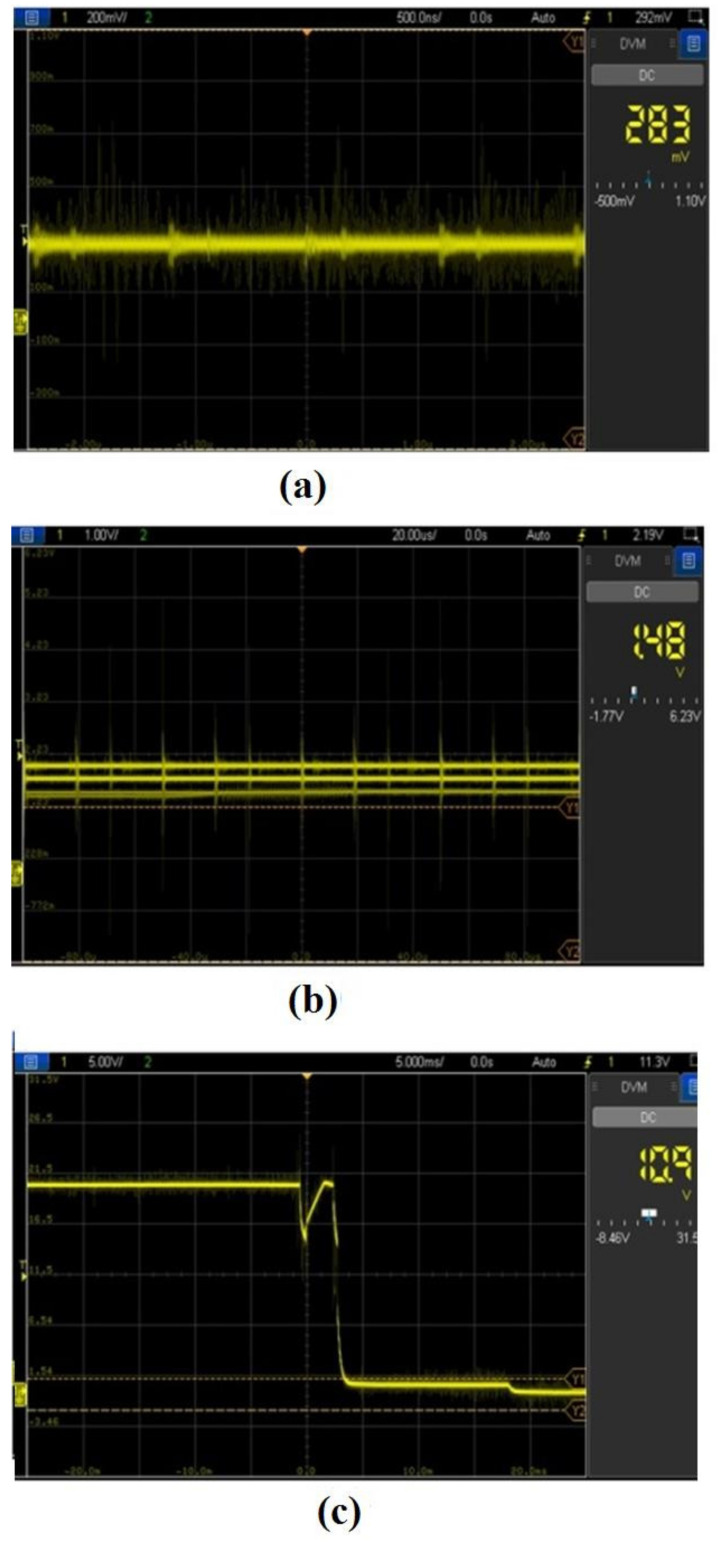
Pulse from analysis during (**a**) short circuit, (**b**) arcing, and (**c**) sparking.

Various waveforms were acquired and recorded using a digital oscilloscope during the machining process in the EDM process. Various phenomena under different distances, such as short circuit, arcing, and sparking, had to be analyzed to enhance the EDM process. Short circuit happens when the voltage across the machining zone is zero. When the distance is a bit high, arcing happens. When a considerable distance is made across the machining zone, a favorable sparking zone happens. Arcing can be converted into sparking by increasing the distance. Figure 6. shows a near short circuit happening across the machining zone. Due to this effect, unwanted disturbances were observed, and an arcing effect was noted, as shown in Figure 6. 

Noises with higher disturbances were viewed owing to the arcing effect. Since a decision was made by signals from the voltage sensor and current sensor, the accuracy could be enhanced considerably. Hence, the proposed method could produce lower signal disturbances compared with those in previous works. It was observed that voltage was reduced at the initiation of the spark in the machining zone. The resolution of a Z-axis linear screw actuation was tested using a high-accuracy ultrasonic sensor. The minimum axis movement was examined to be 0.03 mm. It was also verified using a linear encoder arrangement to check the backlash that happened owing to the proposed mechanism.

### 3.3. Pulse Form Analysis

**Figure 7:**
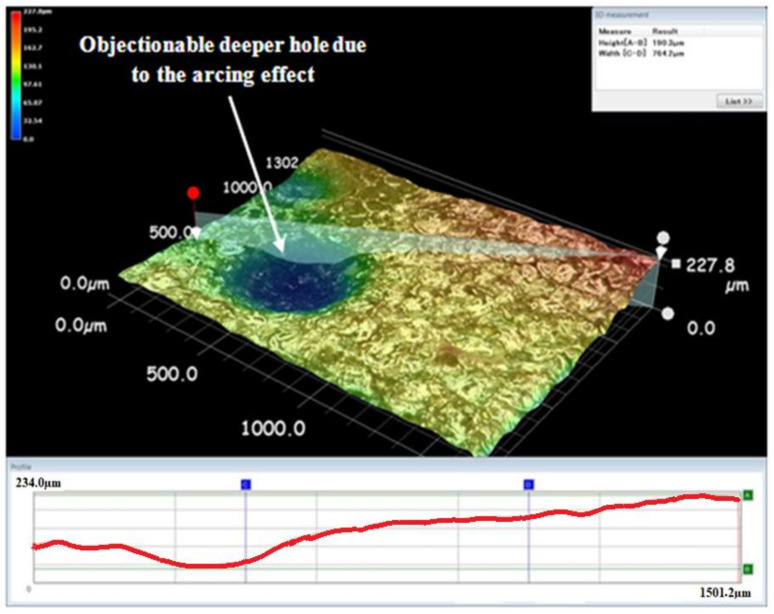
Surface morphology of the machined specimens under arcing.

**Figure 8:**
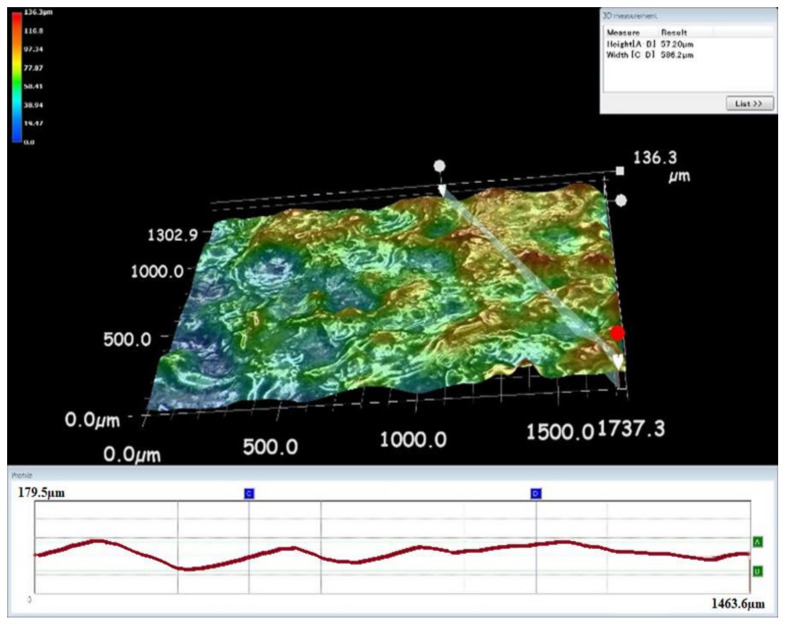
Surface morphology of the machined specimens under sparking.

EDM drilling operations were carried out to determine the effect of the proposed servo tool feed mechanism on the performance metrics of the machining titanium specimens. In this work, surface morphology was used as a performance metric to determine machinability. As seen in Figure 7 and Figure 8, the surface topography of the machined workpiece specimens was collected using a Keyence VHX-5000 microscope.

It was discovered that machining operations are conducted more efficiently when the proposed servo tool feed mechanism is used. However, as illustrated in Figure 7, an undesirable higher crater was detected as a result of the arcing effect. A deeper hole caused by the arcing effect was seen and avoided. The term “sparking” refers to a brief electrical discharge, whereas “arcing” refers to continual sparking. The deeper hole was created across the machined surface as a result of this continuous sparking. In Figure 8, no such unfavorable effect was detected. The proposed approach of the servo feed mechanism in the EDM process significantly improved the material removal rate and surface morphology. Additionally, the proposed gap voltage monitoring-based feed management improved the surface waviness.

### 3.4. Significance of Electrodes on R_a_ while Machining Titanium Specimens

In the EDM process, the spark energy has an effect on the crater size and volume. The lower the spark energy pulses are, the less rough the surface is, whereas the greater the spark energy pulses are, the poorer the surface quality is. Similar and small craters scattered around the surface can help improve the surface’s quality. It was discovered that the electrical conductivity of the tool electrode had a considerable effect on the discharge current determination. Die sinking EDM requires that the die shape is an exact reproduction of the tool electrode at all times. It is recognized that the profile of the workpiece’s surface varies with the tool electrode’s melting point. Because tungsten carbide has a greater melting point and electrical resistivity than other tool electrode materials, such as copper and brass, it can improve the surface quality of the workpiece, as illustrated in Table 3. The electrical resistance of the brass tool electrode is greater than that of the copper tool electrode. Nonetheless, due to its higher melting point than the other electrodes, it cannot create a better surface polish than the copper tool electrode.

### 3.5. Surface Morphology Analysis with Different Tool Electrodes

**Figure 9:**
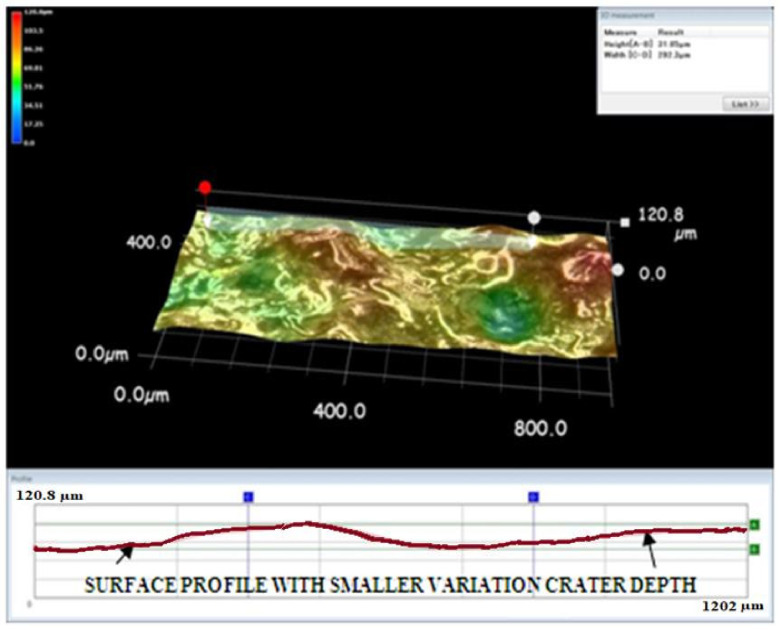
Three-dimensional view of the machined surface using the copper tool electrode.

**Figure 10:**
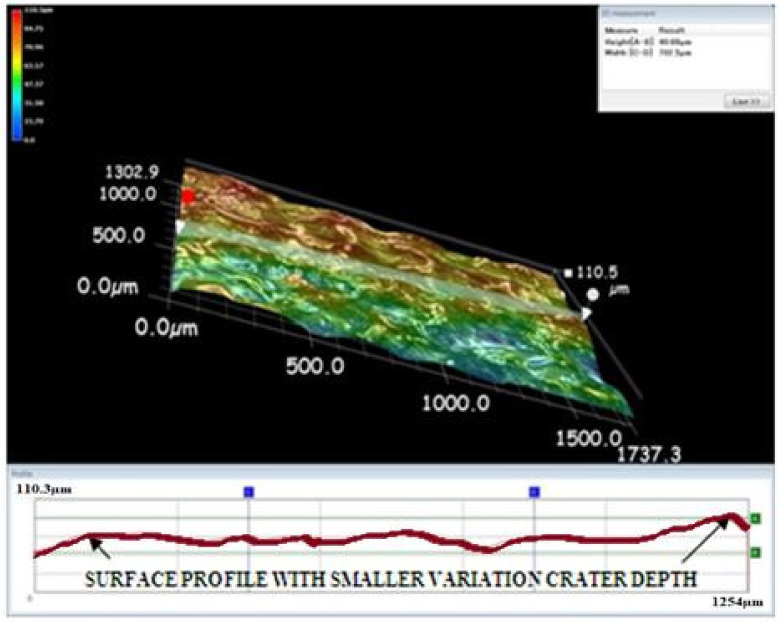
Three-dimensional view of the machined surface using the brass tool electrode.

**Figure 11:**
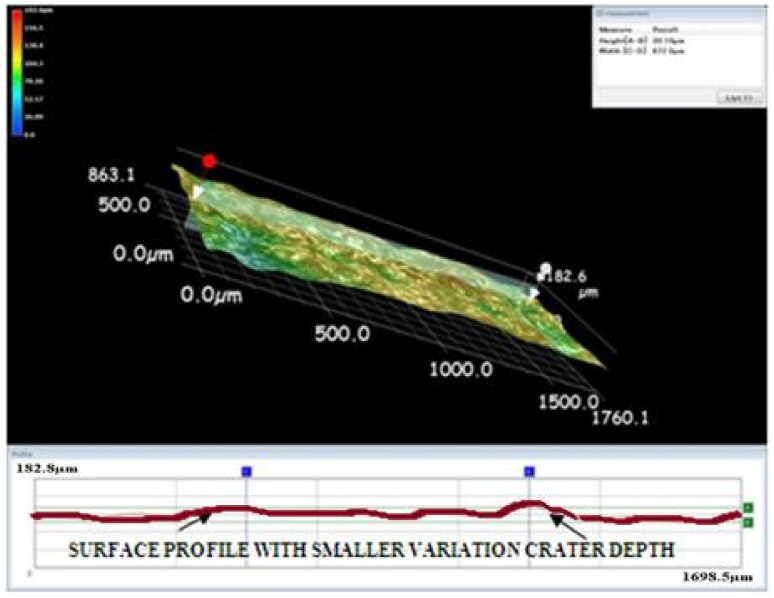
Three-dimensional view of the machined surface using the tungsten carbide tool electrode.

A Keyence microscope was used to obtain three-dimensional views of the machined surface created by the EDM process. The three-dimensional views in Figure 9, Figure 10 and Figure 11 depict the machined surfaces produced by the EDM process utilizing copper, brass, and tungsten carbide tool electrodes, respectively. Due to the tungsten carbide tool electrode’s capacity to generate low energy spark pulses, it produced craters that were smaller in size and volume.

**Figure 12:**
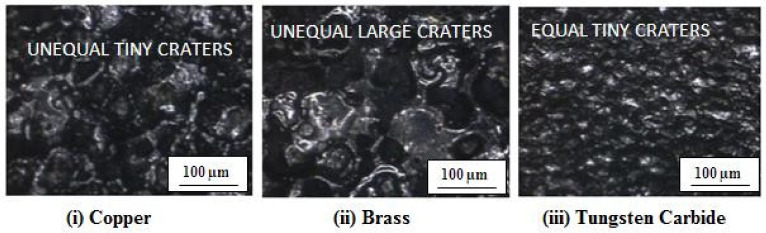
Surface morphology under machining conditions (V = 80 V, I = 15 A, and DF = 0.8).

It is reasonable to conclude that the brass tool electrode produced a higher crater volume with a greater degree of variance in crater size. When the effect of the tool electrodes on the surface finish was considered, it was discovered that the brass tool electrode degraded significantly due to its higher melting point [28,29]. Increased tool electrode degeneration might result in an increase in surface roughness. Because the machined profile on the workpiece was a perfect duplicate of the tool form during the EDM process, the rapidly eroding nature of the brass tool electrode increased the size of the crater. As a result, the brass tool electrode produced a rougher profile of the surface compared with the tungsten carbide and brass tool electrodes. Due to the high melting point of tungsten carbide, it produced a smoother surface compared with other tool electrode materials, such as brass and copper, as shown in Figure 12. Images were taken using a vision measuring system. The tungsten carbide tool electrode could produce a smooth surface due to its tiny and uniform craters. Hence, it could create a lower R_a_. The brass tool electrode could remove the material with larger and uneven craters [30]. Therefore, it could create a higher R_a_ over the specimens during the machining process. 

## 4. Conclusions

The purpose of this work was to design a gap voltage detecting a method for monitoring the servo feed air gap control in the EDM process efficiently. The gap voltage-based servo feed control mechanism suggested in this paper was designed and constructed. Various waveforms were captured and recorded during the EDM machining process using a digital oscilloscope. The following conclusions were drawn from the experimental inquiry.

- The proposed scheme enhances the servo feed mechanism to enhance the machinability as compared with the existing approach due to efficient switching between sparking and arcing.
- The proposed approach on an air gap can produce better surface morphology of the machined specimens.
- The tungsten carbide electrode creates tiny and uniform craters for making a better smooth surface in the EDM process.

## Figures and Tables

**Table 1:** Physical properties of tool electrodes.

Tool Electrode	Electrical Conductivity (S/m)	Melting Point (°C)
Copper	5.96 × 10^7^	1085
Brass	1.67 × 10^7^	930
Tungsten carbide	6.37 × 10^6^	2870

**Table 2:** Process parameters and the variables of the present study.

Trial	V(v)	I(A)	DF
1	40	9	0.4
2	40	9	0.6
3	40	9	0.8
4	40	12	0.4
5	40	12	0.6
6	40	12	0.8
7	40	15	0.4
8	40	15	0.6
9	40	15	0.8
10	60	9	0.4
11	60	9	0.6
12	60	9	0.8
13	60	12	0.4
14	60	12	0.6
15	60	12	0.8
16	60	15	0.4
17	60	15	0.6
18	60	15	0.8
19	80	9	0.4
20	80	9	0.6
21	80	9	0.8
22	80	12	0.4
23	80	12	0.6
24	80	12	0.8
25	80	15	0.4
26	80	15	0.6
27	80	15	0.8

**Table 3:** Influence of tool electrodes on R_a_.

No	Cu	Brass	WC
1.	2.378	3.564	0.384
2.	3.732	4.127	0.474
3.	5.502	7.931	0.612
4.	3.958	5.781	0.482
5.	5.881	6.623	0.627
6.	7.706	9.978	0.742
7.	5.234	7.524	0.591
8.	7.659	10.127	0.733
9.	10.505	14.374	0.878
10.	3.123	3.993	0.408
11.	4.509	5.235	0.579
12.	6.006	8.743	0.654
13.	4.355	6.075	0.545
14.	6.302	7.793	0.689
15.	8.502	10.489	0.804
16.	5.569	7.823	0.607
17.	8.103	10.742	0.779
18.	11.25	15.670	0.912
19.	3.345	4.778	0.415
20.	5.016	5.939	0.601
21.	6.184	9.232	0.685
22.	4.635	6.217	0.588
23.	7.054	9.384	0.707
24.	8.805	12.697	0.838
25.	6.145	8.124	0.678
26.	8.453	10.987	0.807
27.	12.105	17.788	0.925

## Data Availability

The data presented in this study are available from the corresponding author on reason-able request.
